# IRF4 overexpression promotes the transdifferentiation of tregs into macrophage‐like cells to inhibit the development of colon cancer

**DOI:** 10.1186/s12935-021-01766-6

**Published:** 2021-01-19

**Authors:** Jiwei Wang, Song Li, Honglang Li, Xiaoshuang Zhou, Huabin Wen, Bin Lai

**Affiliations:** 1 Department of Ultrasound, The Second Affiliated Hospital of Nanchang University, Nanchang, 330006 Jiangxi China; 2 Mudanjiang Medical College, Mudanjiang, Heilongjiang China; 3 Department of Gastrointestinal Surgery, The Second Affiliated Hospital of Nanchang University, No 1. Minde Road, Nanchang, Jiangxi 330006 China

**Keywords:** IRF4, BCL6, miRNAs, Tregs, Macrophage‐like cells, Colon cancer

## Abstract

**Background:**

Interferon regulatory factor 4 (IRF4) is a transcription factor from the IRF factor family that exerts regulatory functions in the immune system and oncogenesis. However, the biological role of IRF4 in colon cancer is still unclear. The aim of this study is to investigate whether IRF4 participates in the immune response in colon cancer.

**Methods:**

We compared the expression of IRF4, the number of regulatory T cells (Tregs) and macrophages in the colon cancer tissues and paracancerous colon tissues from colon cancer patients. Colon cancer mouse model was established by inoculation with colon cancer cells (SW480) as a xenograft tumor, and we observed tumor growth of colon cancer. Furthermore, the mechanism of action of IRF4 in transdifferentiation of Tregs into macrophage-like cells and the effect of IRF4 on colon cancer cells were investigated *in vitro*.

**Results:**

IRF4 was severely down-regulated in the colon cancer tissues. Colon cancer tissues exhibited an increase in the number of regulatory T cells (Tregs) and macrophages. Furthermore, IRF4 overexpression repressed proliferation, migration and invasion of colon cancer cells (SW480 and HT116 cells). Moreover, IRF4 up-regulation ameliorated tumor growth of colon cancer by promoting the transdifferentiation of Tregs into macrophage-like cells through inhibition of BCL6 expression. Exosomes derived from colon cancer cells repressed IRF4 expression in Tregs by transmitting miR-27a-3p, miR-30a-5p and miR-320c.

**Conclusions:**

IRF4 overexpression promoted the transdifferentiation of Tregs into macrophage-like cells to inhibit the occurrence and development of colon cancer. Thus, IRF4 may be a potential target for colon cancer treatment.

## Background

Colon cancer is a common tumor of the digestive tract, and it is the third most common cause of cancer-related death worldwide. The incidence of colon cancer is increasing year by year and shows younger trend that seriously threatens human health. At present, the treatment of colon cancer has relied mainly on surgery, and supplemented by chemotherapy and radiotherapy. This treatment model has caused a huge burden on the body and mind of patients and national economy. Thus, it is particularly urgent to search new treatments for colon cancer.

In recent years, accumulation stidies have confirmed that the autoimmune system has a crucial role in controlling the progression of tumors by generating antigen-specific immune responses [1, 2]. During the occurrence and development of tumor, an immunosuppressive microecology is increased, which suppresses the anti-tumor immunity of organism and evades the monitoring of the immune system [3]. Regulatory T cells (Tregs) and tumor associated macrophages (TAMs) are generally considered to be involved in the progression of tumors.

During tumor progression, Tregs aggregate and migrate into the tumor microenvironment, and suppress the immune response of anti-tumor cytokines. In the tumor microenvironment, Tregs exhibit a higher levels of inhibitory markers than that in peripheral blood monocytes and non-tumor tissues [4, 5]. Many studies have shown that the accumulation of Tregs in the tumor microenvironment increases with the development of colon cancer [6, 7]. Furthermore, TAMs play a crucial role in tumorigenesis by secreting a series of cytokines, growth factors, and infectious factors. 80 % of TAMs show anti-inflammatory effects similar to M2 macrophages, but promote the formation of tumor. A small proportion of TAMs show pro-inflammatory effects similar to M1 type macrophages, but inhibits tumor growth [8, 9]. Moreover, the increase of M2 macrophages is positively correlated with the progression of colon cancer [10]. Thus, TAMs more exhibit M1 macrophage-like effects may inhibit tumor development.

Tregs are unstable and can be transformed into various effector T cells such as Th1, Th2, Th17 and Tfh cells under the mediation of transcription factors T-bet, interferon regulatory factor 4 (IRF4), STAT3, BCL6 [11–14]. The transformation characteristic of Tregs is called the plasticity of Tregs. However, whether Tregs can be transformed into “macrophage-like” cells has not been reported. Our previous research has found that knockout of transcription factor BCL6 causes an increase of macrophages in Tregs, especially M1 macrophages. The transformation of Tregs into macrophage-like cells is called “a new feature of Tregs plasticity”.

BCL6 is a transcriptional repressor and participates in regulating the germinal center. BCL6 is also a frequently activated oncogene in the pathogenesis of human B-cell lymphoma [15]. Moreover, BCL6 takes part in the inflammatory activity and differentiation of Th2 cells [16, 17]. In addition, IRF4 plays a role in the regulation of Treg differentiation in human cancer [18]. IRF4 regulates the immunosuppressive function of Tregs in primary immune thrombocytopenia [19]. IRF4 participates in the regulation of immune response and the development metabolism of immune cells [20]. More importantly, IRF4, as an upstream transcription factor of BCL6, negatively regulates the expression of BCL6 [21]. Tregs have been reported to mediate the immunosuppressive response of Tregs through the non-cell autonomous regulation of exosomes containing miRNAs [22, 23]. Our previous research has also found that exosomes secreted by colon cancer cells contains many microRNAs (miRNAs), including miR-320c, miR-27a-3p and miR-30a-5p. These miRNAs bind to IRF4 in Tregs and inhibit the activity of IRF4. Inhibition of IRF4 enhances the expression of BCL6, thereby stabilizing the level of Tregs and suppressing the anti-tumor immune response.

Based on the new features of Tregs transformation, we speculated that overexpression of IRF4 inhibited the expression of BCL6, which reduced the stability of Tregs and promoted the transformation of Tregs into “macrophage-like” cells. The reduction of Tregs weakened the anti-tumor immunosuppression, and the transformed macrophage-like cells (especially M1 macrophages) may play an anti-tumor effect, thereby synergistically inhibiting the proliferation of colon cancer cells.

## Materials and methods

### Clinical specimen collection

Colon cancer tissue and paracancerous colon tissue specimens were collected from 24 colon cancer patients who underwent radical resection of colon cancer at The Second Affiliated Hospital of Nanchang University. The tumor tissue specimens were diagnosed as colon cancer by rapid frozen pathological examination during the operation and routine pathological examination. The colon cancer tissues and paracancerous colon tissues were rapidly snap-frozen in liquid nitrogen. The tissues were stored at − 80 °C for further analysis. Patients who received radiochemotherapy or molecular biological treatment were excluded. The participants were informed and gave written consent. All protocols were authorized by the Ethics Committee of The Second Affiliated Hospital of Nanchang University.

### Cell culture

Human colon cancer cells (SW480 and HCT116) were obtained from public cell banks (ATCC, Manassas, VA, USA). SW480 and HCT116 cells were cultured in RPMI 1640 medium (Sangon Biotech, Shanghai, China) supplemented with 10 % fetal bovine serum (FBS, Gibco, Middleton, WI, USA) and 1 % penicillin/streptomycin (Sangon Biotech). These cells were incubated in a humidified atmosphere at 37 °C and 5 % CO_2_.

### Plasmids and transfectionPlasmids and transfection

Full length of IRF4 or BCL6 was cloned into the lentivirus vector Ubi-MCS-3 FLAG-CBh-gcGFP-IRES-puromycin, and then packaged into lentivirus (RIBOBIO, Guangzhou, China). For knockdown of IRF4, shRNA specifically targeting IRF4 (shRNA: ccgg-GCC CAA ATT CTC CTC TCT AAA-ctcgag-TTT AGA GAG GAG AAT TTG GGC-ttttt) was cloned in the lentiviral vector pLKO-Puro (LV-shRNA) (RIBOBIO). The miR-320c mimic (miR-320c mimic: 5’-AAA AGC UGG GUU GAG AGG GUC CUC UCA ACC CAG CUU UUU U-3’; miR-320c mimic NC: 5’-UUC UCC GAA CGU CUC ACG UTT ACG UGA CAC GUU CGG AGA ATT-3’), miR-27a-3p mimic (miR-27a-3p mimic: 5’-UUC ACA GUG GCU AAG UUC CGC-3’; miR-27a-3p mimic NC: 5’-UUU GUA CUA CAC AAA AGU ACUG-3’), miR-30a-5p mimic (miR-30a-5p mimic: 5’-GCU UCC AGU CGA GGA UGU UUA CA-3’; miR-30a-5p mimic NC: 5’-CAG UAC UUU UGU GUA GUA CAA-3’), miR-320c inhibitor (miR-320c inhibitor: 5’-ACC CUC UCA ACC CAG CUU UU-3’; miR-320c inhibitor NC: 5’-CAG UAC UUU UGU GUA GUA CAA-3’), miR-27a-3p inhibitor (miR-27a-3p inhibitor: 5’-GCG GAA CUU AGC CAC UGU GAA-3’; miR-27a-3p inhibitor NC: 5’-CAG UAC UUU UGU GUA GUA CAA A-3’), miR-30a-5p inhibitor (miR-30a-5p inhibitor: 5’-CUU CCA GUC GAG GAU GUU UAC A-3’; miR-30a-5p inhibitor NC: 5’-CAG UAC UUU UGU GUA GUA CAA-3’) and the corresponding NC were synthesized by RIBOBIO. Plasmids were transfected into the cells using Lipofectamine 2000 (Invitrogen, Carlsbad, CA, USA) following the manufacturer’s protocol.

### Experimental animals

BALB/c mice with 6–8 weeks old were purchased from Beijing Vital River Laboratory Animal Technology Co., Ltd. (Beijing, China). These mice were housed in a standard laboratory environment (21 ± 1 °C; 45–55 % humidity; 12 h light/12 h dark cycle; free access to feed and water). Colon cancer mouse model was established by subcutaneous injection with SW480 cells (1 × 10^6^ cells/200 µL) into mouse through the right armpit. BALB/c mice were randomly divided into 5 groups. Model group: mice were subcutaneously injected with SW480 cells; LV-ctrl group: mice were subcutaneously injected with LV-ctrl-transfected SW480 cells; LV-IRF4 group: mice were subcutaneously injected with LV-IRF4-transfected SW480 cells; LV-shRNA group: mice were subcutaneously injected with LV-shRNA-transfected SW480 cells; LV-sh-IRF4 group: mice were subcutaneously injected with LV-sh-IRF4-transfected SW480 cells. SW480 cells were transfected with LV-IRF4, LV-ctrl, LV-sh-IRF4 or LV-shRNA to obtain the transfected SW480 cells.

### Quantitative real‐time PCR (qRT-PCR)

QRT-PCR was used to measure the expression of IRF4, BCL6, miR-320c, miR-27a-3p and miR-30a-5p in the cells and tissues. TRIzol reagent (Invitrogen) was used to extract total RNA from cells or tissues as the protocol described. The purity and concentration of RNA was detected using NanoDrop 2000 spectrophotometer (Thermo Fisher Scientific, Waltham, MA, USA). RNA integrity was examined by 1.5 % agarose gel electrophoresis. The RNA was reversely transcribed to complementary DNA using PrimeScript™ RT Reagent Kit with gDNA Eraser (Takara, Tokyo, Japan). The relative expression of genes was assessed by performing qRT-PCR using SYBR Green PCR Mix Kit (Takara) on a Real-Time PCR Instrument (Applied Biosystems, Carlsbad, CA, USA). RNA and water with no sample were used as negative controls in the qRT-PCR to identify gDNA contamination and general contamination. Housekeeping gene GAPDH was used as a reference gene for normalization. PCR reactions were performed as these conditions: step 1 (denaturation): 95 °C, 5 min; step 2 (amplification): 95 °C for 10 s and 60 °C for 30 s, 45 cycles; step 3 (cooling): 40 °C, 30 s. The results were analyzed using the 2^−∆∆CT^ (cycle threshold) method. Primer sequences were as follows: IRF4: forward: 5′-AGA CTG TGC CAG AGC AGG AT-3′, reverse: 5′‐GGG TCT GGA AAC TCC TCT CC‐3′; BCL6: forward: 5′‐TTC CGC TAC AAG GGC AAC-3′, reverse: 5′-TGC AAC GAT AGG GTT TCT CA-3′; miR-27a-3p: forward: 5′-CAT CTG AGG ATT CAC AGT GGC TA-3′, reverse: 5′-CTC AAC TGG TGT CGT GGA GTC-3; miR-320c: forward: 5′-AAA AGC AGG GAA GAG AGG GA -3′, reverse: 5′-ATT CCA TGA GAG ATC CCT AGC GT-3′; miR-30a-5p: forward: 5′-TGT AAA CAT CCT GCA C-3′, reverse: 5′-ACA TCC AGT GTA GCA TA -3′; GAPDH: forward: 5′-GGG AGC CAA AAG GGT CAT-3′, reverse: 5′-GAG TCC TTC CAC GAT ACC AA-3′.

### Western blot (WB)

Total protein was extracted from cells or tissues using Tissue or Cell Total Protein Extraction Kit (Sangon Biotech) following the protocol of manufacturer. Equivalent protein (25 µg) from different samples was separated by 10 % SDS-PAGE protein electrophoresis. The separated protein samples were transferred onto the PVDF membranes (Merck Millipore, Billerica, MA, USA). Then, the membranes were incubated with 5 % skim milk to block the non-specific sites. After that, the membranes were incubated with the primary antibodies, anti-rabbit IRF4 or anti-rabbit BCL6 (1:1000, Cell Signaling Technology, Danvers, MA, USA) at 4 °C for 12 h. After the membranes were washed with TBST for several times, the membranes were stained with horseradish peroxidase-conjugated second antibody, goat anti-rabbit IgG antibody (1:1000, Cell Signaling Technology) at room temperature for 1 h. Anti-rabbit β-actin antibody (1:1000, Cell Signaling Technology) was used as a reference protein for normalization. The gray levels of the protein bands were examined by Image J software.

### Immunohistochemistry

The colon cancer tissues and adjacent tissues were fixed in 4 % paraformaldehyde and embedded in paraffin. Four-micron sections were obtained after deparaffin and rehydration. The sections were heated for 20 min in 10 mM sodium citrate for antigen retrieval. Endogenous peroxidase activity was blocked by incubating the tissue with 3 % hydrogen peroxidase in methanol for 5 min. The sections were incubated with primary polyclonal antibodies, Foxp3 (1:100, eBioscience, Cambridge, UK), CD86 (1:100, eBioscience) or CD206 (1:1000, eBioscience) for 15 min at room temperature. Then, the sections were incubated with a horseradish peroxidase-conjugated secondary antibody (Maxim-Bio, Fuzhou, China) for 30 min. Labeling was monitored by 3, 3’-diaminobenzidine chromogen (Maxim-Bio). At last, hematoxylin was used to stain the sections, and the sections were observed under an optical microscope at 400 times magnification.

### Hematoxylin‐eosin (HE) staining

Colon cancer tissues of mice were fixed in 4 % paraformaldehyde and embedded in paraffin. Five-micron sections were obtained after deparaffin and rehydration. Then, sections were stained using HE staining kit (Solarbio, Beijing, China) as the protocol described. The pathological changes of tumor tissues were observed under an optical microscope at 400 times magnification.

### Flow cytometry analysis of tregs and macrophages

The colon cancer tissues of mice were cut into 1–2 mm^3^ pieces, and then grinded into homogenate. The cell suspension was filtered through a 70 µm nylon filter and washed with PBS for several times. Cell suspension of colon cancer tissues was used to assess the proportions of Tregs and macrophages in the colon cancer tissues. The differentiated Tregs were used to examine the proportions of macrophages in the Tregs. For the proportions of Tregs, cell suspension (100 µL) of colon cancer tissues or Tregs at concentration of 1 × 10^7^ cells/mL were stained with CD4-APC (ab18280, Abcam) and CD25-FITC (ab210332, Abcam) antibodies for 30 min at 4 °C in dark. After washed with PBS, the cell suspension was incubated with 1 µL fixation/permeabilization membrane breaker at room temperature for 40 min. The cell suspension was washed with membrane breaking buffer and incubated with 10 µL Foxp3-PE (ab210231, Abcam) antibody in darkness for 30 min. Subsequently, the cell suspension was suspended with 500 µL PBS. For the proportions of macrophages, cell suspension was stained with 10 µL CD86-FITC (ab234237, Abcam) or CD206-FITC (ab270647, Abcam) and F4/80-APC (ab105080, Abcam) antibodies. Filally, the proportions of Tregs or macrophages in colon cancer tissues and the proportions of macrophages in Tregs were detected using a FACSCalibur (BD Biosciences, San Jose, CA, USA).

### Differentiation and treatment of tregs

Peripheral blood mononuclear cells (PBMC) were isolated from peripheral blood of colon cancer patients by density-gradient centrifugation with Ficoll. Then, CD4^+^ T cells were separated from PBMC using Dynabeads™ CD4 Positive Isolation Kit (Thermo Fisher Scientific) according to the manufacturers’ instruction. The purity of the sorted CD4^+^ T was verified by flow cytometry. CD4^+^ T cell suspension was stained with CD4-FITC (Abcam) at 4 °C for 30 min. Then, CD4^+^ T cells were gated with CD4 and SSC, the purity of CD4^+^ T cells was examined using a FACSCalibur (BD Biosciences).

CD4^+^ T cells and Tregs were cultured in RPMI 1640 medium containing 10 % FBS and 1 % penicillin/streptomycin at 37 °C and 5 % CO_2_. For activation of CD4^+^ T cells, sorted naive CD4^+^ T cells were activated with anti-CD3 and anti-CD28 (BD Biosciences). For differentiation of Tregs, CD4^+^ T cells were incubated with TGF-β (5.0 ng/mL) for 5 days. Tregs were stained with CD25-FITC and Foxp3-PE antibodies to estimate the Treg differentiation efficiency by flow cytometry. Subsequently, Tregs were transfected with LV-IRF4, LV-ctrl, LV-sh-IRF4 or LV-shRNA. Then, the cell culture medium of these modified Tregs was collected by centrifugation, and then incubated with SW480 or HCT116 cells.

### Phagocytosis

The phagocytosis assay was performed using 1 µm fluorescent beads (Fluoresbrite® Yellow Green Microspheres, Polysciences, Eppenheim, Germany) as the protocol described. For the experiment, 5 × 10^5^ cells were incubated with 1 × 10^7^ beads for 2 h. Then, the cells were stained with DAPI at room temperature for 10 min. The levels of engulfed beads of the cells were detected using a FACSCalibur (BD Biosciences) with CellQuest software.

### MTT assay

MTT assay was performed to estimate cell proliferation. The cells at log phase were seeded into 96-well plates at a density of 5 × 10^3^/well. After that, MTT reagent (20 µL) was added into each well, and the cells cultured for 4 h in an incubator at 37 °C. Then, supernatant was discarded and DMSO reagent (100 µL) was added into each well. The absorbance of samples was detected at 570 nm wavelength using enzyme-labeled instrument (Thermo Fisher Scientific).

### Wound‐healing assay

The cells were seeded in a six-well plate at a density of 1 × 10^6^/well. Then, the cells were scratched with a 20-µL pipette tip after the cell density reached 80 % or 90 %. The cells were washed with PBS for 3 times to remove the cell debris, and then cultured in serum-free RPMI 1640 medium at 37 °C and 5 % CO_2_ for 12 h. Wounds were observed by microscopy and photographed at 0, 24 and 48 h after wounding. The wound areas were calculated using Image J software.

### Transwell invasion assay

The transwell invasion assay was performed using a 24-well Boyden chamber with 8 µm pore size polycarbonate membrane (Corning, NY, USA). Matrigel was diluted to 1 mg/mL with serum-free medium and covered on the upper chamber of Boyden chambers. Then, the cell suspension was seeded into the upper chamber; 600 µL fresh culture medium containing 10 % FBS was added into the lower chamber. After cultured at 37 °C for 24 h, invading cells on the bottom surface of the chamber were fixed with 4 % paraformaldehyde and stained with 0.5 % crystal violet. The invasive cells were observed and counted under an Olympus fluorescence microscope (Tokyo. Japan).

### Chromatin immunoprecipitation (ChIP)

ChIP assay was performed to analyze the interaction between IRF4 and BCL6 promoter using EpiQuik™ Chromatin Immunoprecipitation Kit (EpiGentek, Farmingdale, NY, USA) according to the manufacturer’s instructions. Briefly, the cells were cross-linked with formaldehyde and then homogenized. The homogenate was sonicated to generate short fragments of genomic DNA. Then, equal amounts of treated chromatin were added to microwells containing immobilized antibody for the targeted protein IRF4. Cross-linked DNA is released from the antibody-captured protein-DNA complex, reversed, and purified through the Fast-Spin Column. The purified DNA was used for PCR analysis.

### Transwell co‐culture

SW480 cells and Tregs were co-cultured in a 6-well transwell chamber (Corning) with 0.4 µm porous membrane. Subsequently, Tregs were seeded in the lower chamber and SW480 cells were seeded in the upper chamber. Cells were cultured in RPMI 1640 medium. SW480 cells were incubated with 10 µM GW4869 to inhibit the generation of exosoems.

### Isolation and treatment of exosomes

Cells were grown to the third generation in RPMI 1640 medium without exosomes. The supernatant of culture was collected by centrifugation at 3000*g* for 15 min to remove cell debris, and then filtrated by 0.22 µm ultrafiltrate membrane under aseptic conditions. Next, the liquid was concentrated by ultrafiltration tube. The exosomes were extracted by Total Exosome Isolation Reagent Kit (from cell culture media) (Invitrogen) as the introduction described. Then, the exosomes were incubated with Tregs.

### Luciferase reporter assay

IRF4 containing the predicted miR-320c, miR-27a-3p or miR-30a-5p binding sites were cloned into pGL3-IRF4-Wt (wild type), pGL3-IRF4-Mut (mutant type) vectors (RIBOBIO) respectively. The miR-320c mimic, miR-27a-3p mimic, miR-30a-5p mimic and the corresponding mimic NC were synthesized by RIBOBIO. The Wt (Mut) 3′untranslated region (UTR) of IRF4 vector and miR-320c mimic, miR-27a-3p mimic, miR-30a-5p mimic or the corresponding mimic NC were co-transfected into 293 cells by using Lipofectamine 2000 Transfection Reagent (Invitrogen). pRL-TK vector was transfected into 293 cells as a reference for normalization. After 48 h of transfection, Dual luciferase assay kit (Promega, Madison, USA) was used to measure the activities of firefly and renilla luciferase on luciferase assay system (Ambion, Austin, TX, USA). The relative Rluc/Luc ratio was calculated to analyze the relationship among IRF4, miR-320c, miR-27a-3p and miR-30a-5p.

### Statistical analysis

All experiments were independently repeated at least 3 times. All values were exhibited as mean ± standard deviation and analyzed by SPSS 22.0 statistical software (IBM, Armonk, NY, USA). For comparison of two groups, a two-tailed Student’s t test was used. Comparison of multiple groups was made using a one- or two-way ANOVA. Difference was considered statistically significant at *P* < 0.05.

## Results

### IRF4 was down‐regulated in colon cancer tissues

**Fig. 1:**
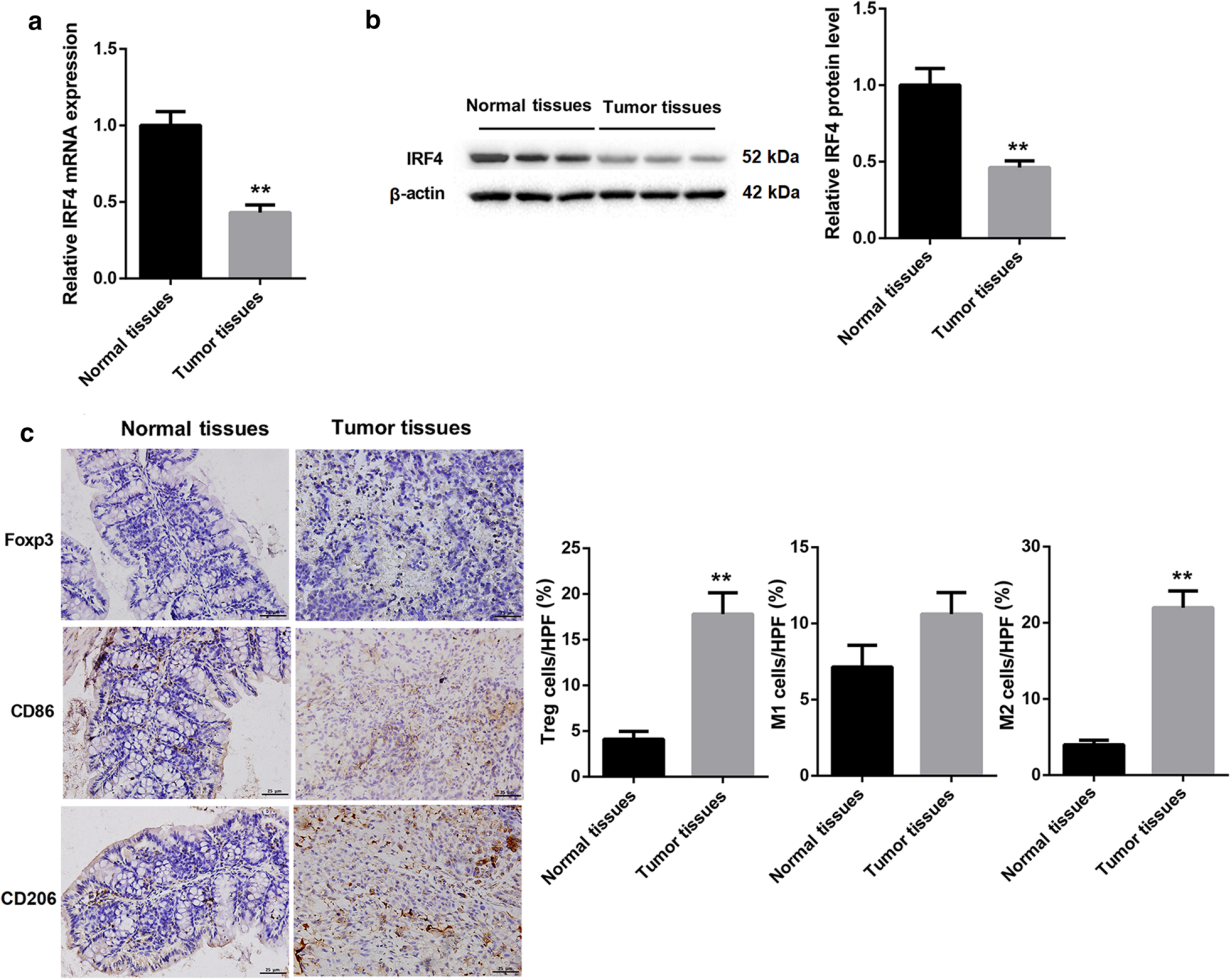
IRF4 was down-regulated in colon cancer tissues. Colon cancer tissue and paracancerous colon tissue specimens were collected from colon cancer patients. QRT-PCR (**a**) and WB (**b**) were performed to assess the gene and protein expression of IRF4 in colon cancer tissues and paracancerous colon tissues. **c** Immunohistochemistry was performed to detect the proportions of Tregs and macrophages in colon cancer tissues and paracancerous colon tissues. (**P < 0.01, versus Normal adjacent tissues)

To disclose differentially expressed IRF4 in colon cancer, we analyzed IRF4 expression in colon cancer tissues and paracancerous colon tissues from colon cancer patients. The data obtained from qRT-PCR and WB showed that IRF4 was highly expressed in colon cancer tissues with respect to the normal paracancerous colon tissues (Fig. 1a, b). Additionally, we estimated the proportions of Tregs and macrophages in colon cancer tissues and paracancerous colon tissues. We found that colon cancer tissues exhibited an increase in the proportions of Tregs and M2 macrophages. Compared with normal paracancerous colon tissues, the proportions of M1 macrophages was slightly elevated in colon cancer tissues. However, there was no significant difference in the proportions of M1 macrophages between colon cancer tissues and paracancerous colon tissues (Fig. 1c). Thus, these data indicated that down-regulation of IRF4 was associated with colon cancer.

### IRF4 overexpression attenuated colon cancer damage and affected the level of Tregs in colon cancer tissues 

**Fig. 2:**
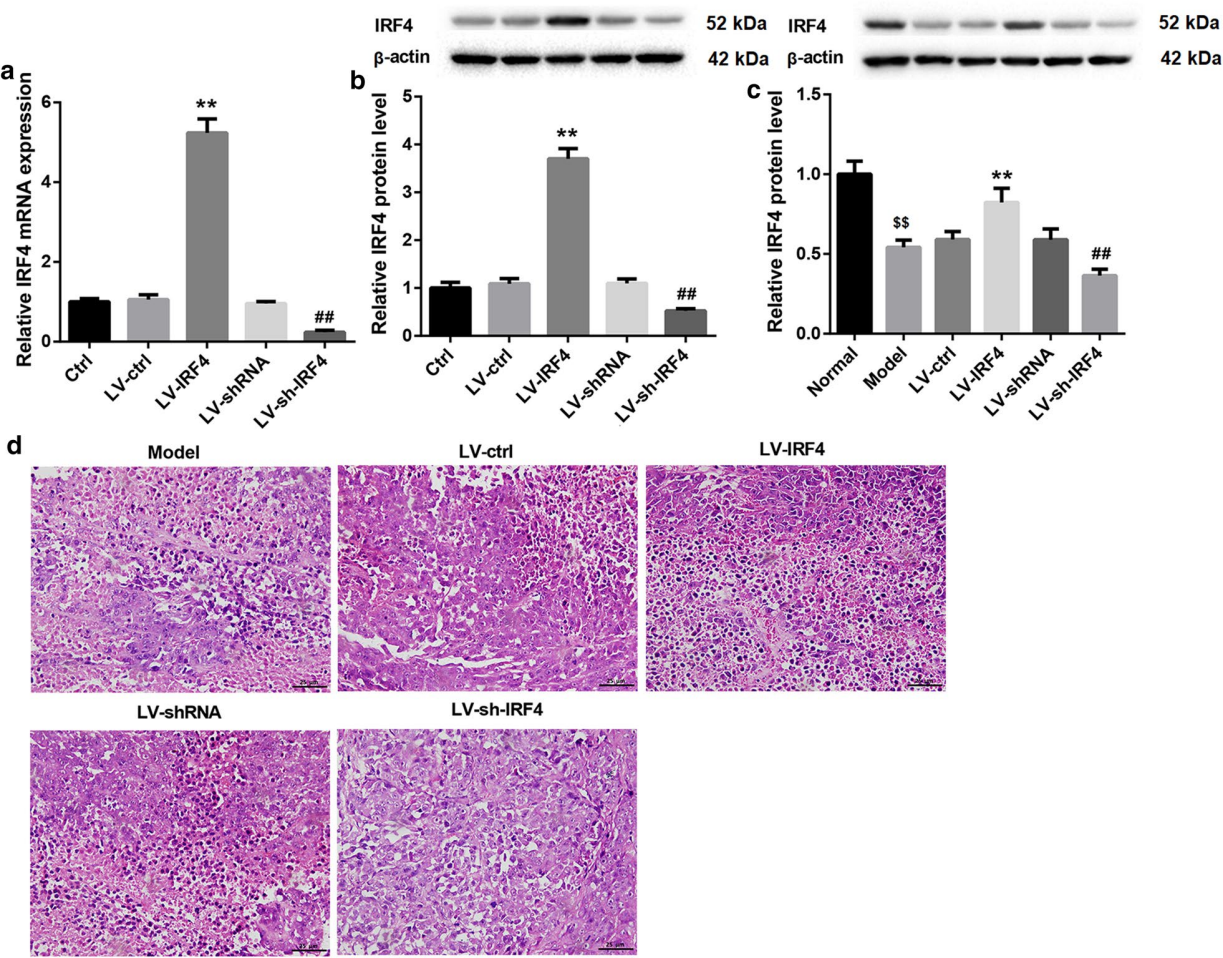
IRF4 overexpression attenuated colon cancer damage. SW480 cells were transfected with LV-IRF4, LV-ctrl, LV-sh-IRF4 or LV-shRNA. Normal SW480 cells served as control. QRT-PCR (**a**) and WB (**b**) were performed to assess the gene and protein expression of IRF4 in the modified SW480 cells. BALB/c mice were injected with normal or the modified SW480 cells. **c** The expression of IRF4 in the tumors of mice was measured by qRT-PCR. The colon tissues form normal BALB/c mice served as control. **d** HE staining was performed to explore the pathological changes of tumors. (^$$^*P* < 0.01, versus Normal; *P < 0.05, **P < 0.01, versus LV-ctrl; ^#^*P* < 0.05, ^##^*P* < 0.01, versus LV-shRNA)

**Fig. 3:**
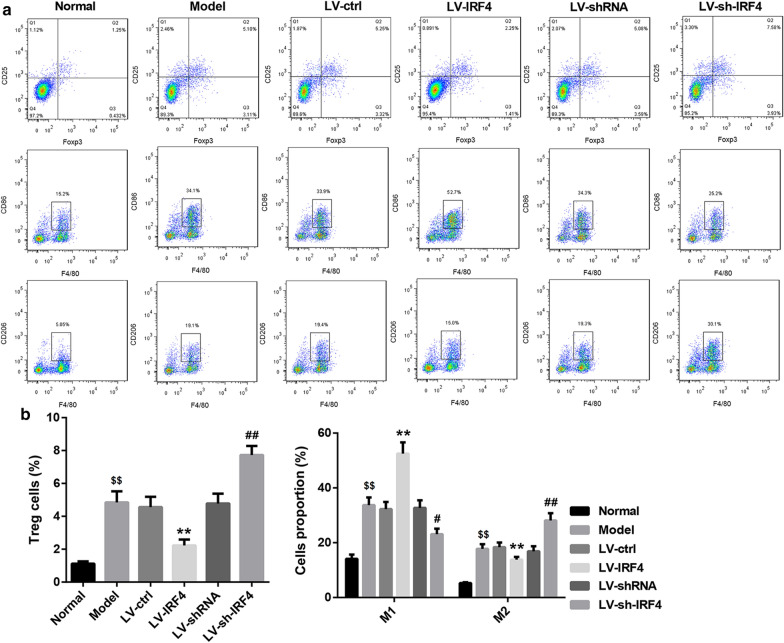
IRF4 overexpression reduced Tregs and M2 macrophages and enhanced M1 macrophages in the colon cancer. SW480 cells were transfected with LV-IRF4, LV-ctrl, LV-sh-IRF4 or LV-shRNA. Then, BALB/c mice were injected with normal or the modified SW480 cells. Normal BALB/c mice served as control. **a**, ** b** The proportions of Tregs and macrophages in the tumor tissues of mice were detected by flow cytometry. The colon tissues form normal BALB/c mice served as control. (^$$^*P* < 0.01, versus Normal; **P < 0.01, versus LV-ctrl; ^#^*P* < 0.05, ^##^*P* < 0.01, versus LV-shRNA)

To explore the biological role of IRF4 in colon cancer, we overexpressed or knocked down IRF4 in SW480 cells by transfecting them with LV-IRF4 or LV-sh-IRF4. The results of qRT-PCR and WB showed that IRF4 overexpression caused an up-regulation of IRF4, while the gene and protein expression of IRF4 in SW480 cells was decreased in the presence of LV-sh-IRF4 (Fig. 2a, b). To further examine the function of IRF4 *in vivo*, we subcutaneously injected SW480 cells with stable overexpression or knockdown of IRF4 into the BALB/c mice. IRF4 was severely down-regulated in the tumor tissues of Model group as compared with the normal colon tissues. LV-IRF4 group displayed an up-regulation of IRF4 as compared with LV-ctrl group. The expression of IRF4 was lower in LV-sh-IRF4 group than that in LV-shRNA group (Fig. 2c). We also found that the colon cancer tissues in Model group displayed a significant damage. The colon cancer tissue damage was attenuated in LV-IRF4 group and aggravated in LV-sh-IRF4 group (Fig. 2d). Moreover, we estimated the proportions of Tregs and macrophages in the colon cancer tissues of mice by flow cytometry. Compared with normal colon tissues, the tumor tissues from Model group exhibited a boost in the proportions of Tregs, M1 and M2 macrophages. The proportions of Tregs and M2 macrophages were decreased, whereas the proportions of M1 macrophages were elevated in LV-IRF4 group. Compared with LV-shRNA group, LV-sh-IRF4 group displayed an increase in the proportions of Tregs and M2 macrophages, and exhibited a decrease in the proportions of M1 macrophages (Fig. 3a, b). Therefore, these results suggested that IRF4 overexpression attenuated colon cancer damage and regulated the level of Tregs in colon cancer tissues.

### IRF4 overexpression promoted the transdifferentiation of Tregs into macrophage-like cells and inhibited cell proliferation, migration and invasion of colon cancer cells 

**Fig. 4:**
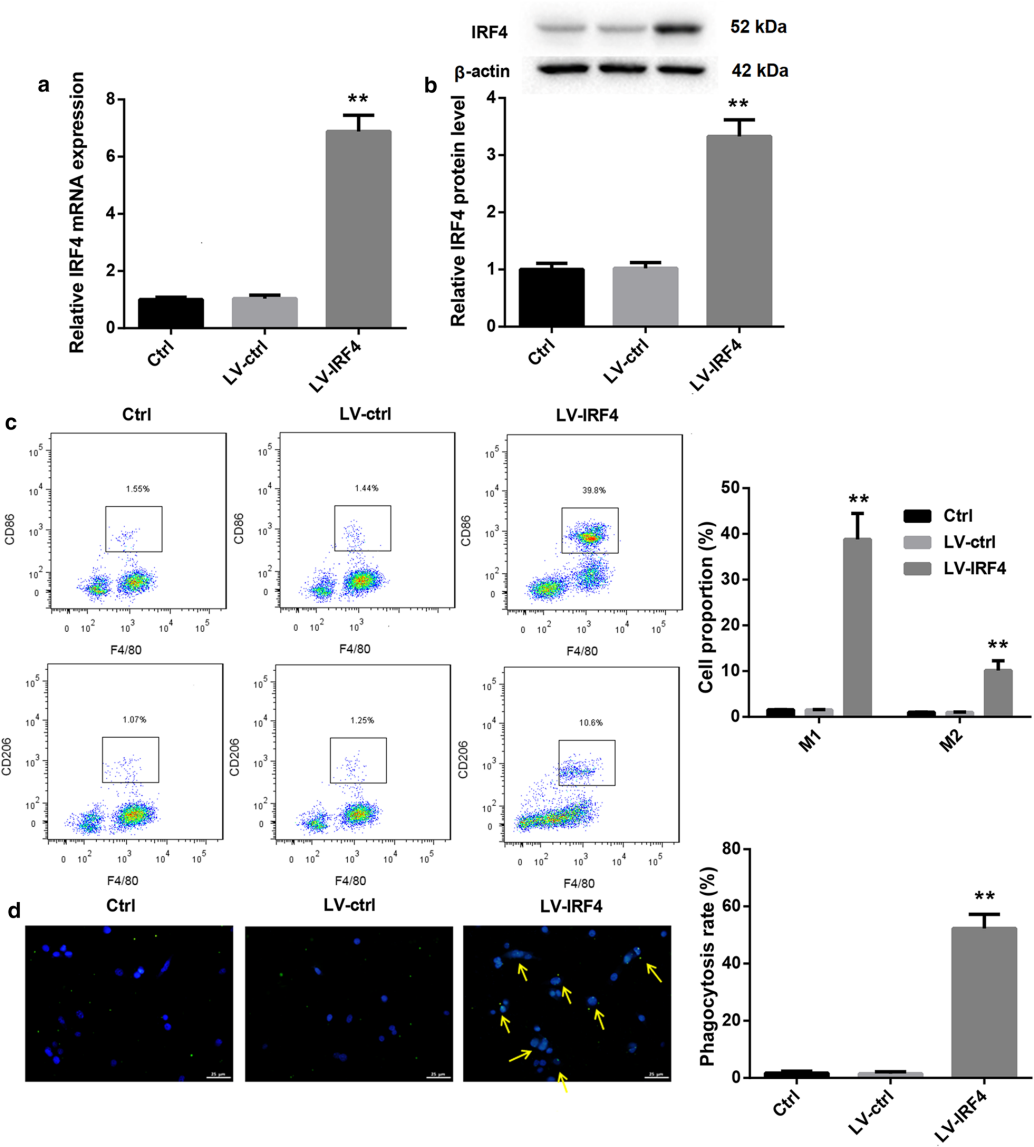
IRF4 overexpression promoted the transdifferentiation of Tregs into macrophage-like cells. Tregs were transfected with LV-IRF4 or LV-ctrl. Normal Tregs served as control. QRT-PCR (**a**) and WB (** b**) were performed to assess the gene and protein expression of IRF4 in the modified Tregs. **c** The proportions of macrophages in the modified Tregs were detected by flow cytometry.** d** The phagocytosis assay was performed to assess the phagocytosis of the modified Tregs. The phagocytosis positive cells were marked by arrows. (**P < 0.01, versus LV-ctrl)

**Fig. 5:**
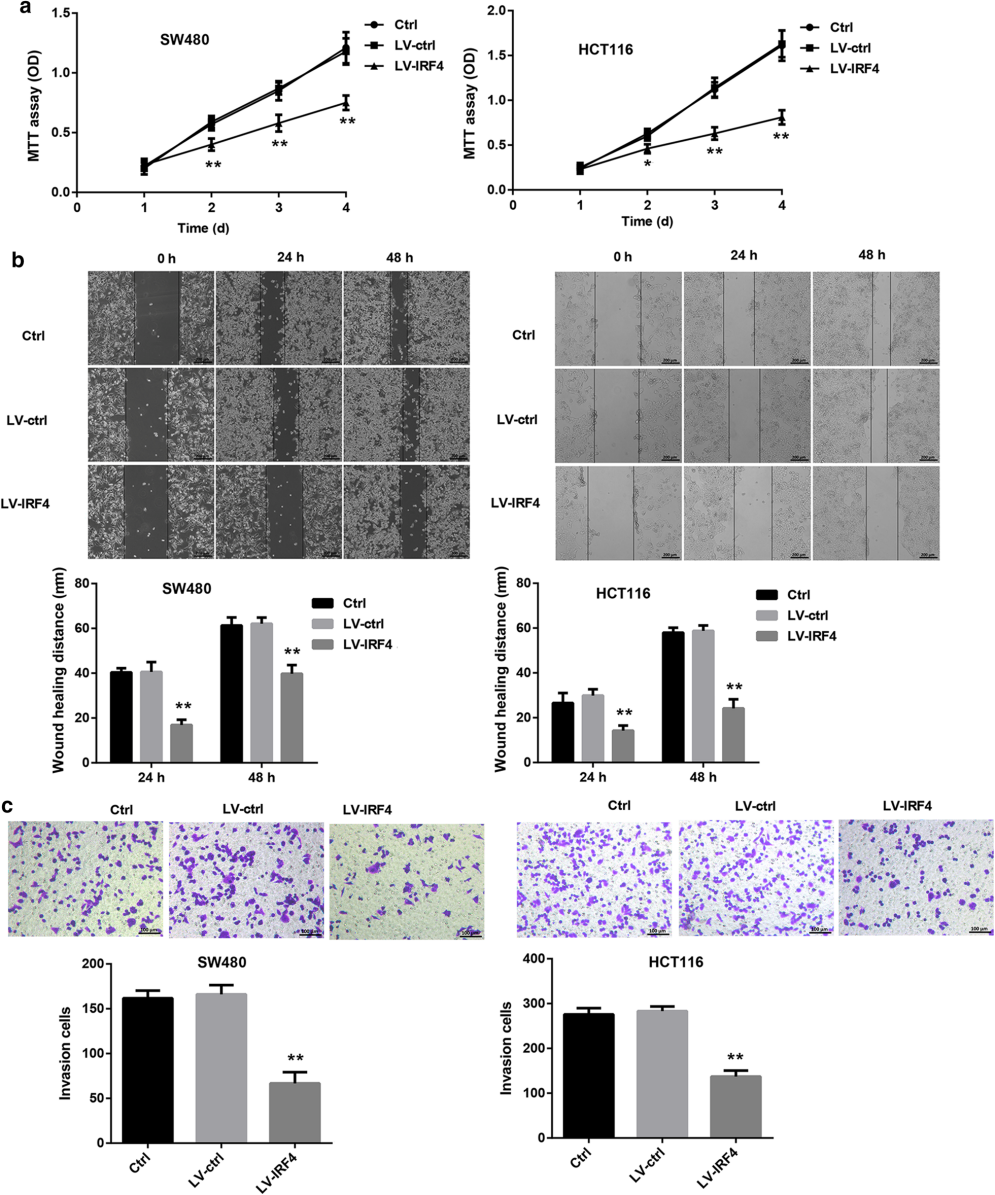
IRF4 overexpression repressed cell proliferation, migration and invasion of SW480 and HCT116 cells. Tregs were transfected with LV-IRF4 or LV-ctrl. Normal Tregs served as control. SW480 and HCT116 cells were incubated with the cell culture medium of the normal or modified Tregs. **a** MTT assay was performed to estimate cell proliferation of SW480 and HCT116 cells. **b** Wound-healing assay was performed to assess migration of SW480 and HCT116 cells. **c** Transwell invasion assay was performed to explore invasion of SW480 and HCT116 cells. (*P < 0.05, **P < 0.01, versus LV-ctrl)

Next, we explored whether IRF4 participated in the transdifferentiation of Tregs in colon cancer. We first separated CD4^+^ T cells from PBMC of colon cancer patients, and the isolated CD4^+^ T cells always remained above 90 % (Additional file 1: Fig. S1). Tregs were differentiated from activated CD4^+^ T cells, and the Treg differentiation efficiency reached about 75 % (Additional file 2: Fig. S2). Then, Tregs were transfected with LV-IRF4 to induce IRF4 up-regulation, and the gene and protein expression of IRF4 in Tregs was significantly enhanced in the presence of LV-IRF4 (Fig. 4a, b). Furthermore, we estimated the influence of IRF4 overexpression on macrophage polarization by flow cytometry, showing that IRF4 up-regulation led to an increase in the proportions of M1 and M2 macrophages in Tregs (Fig. 4c). We also found that that IRF4 up-regulation notably promoted phagocytosis of Tregs (Fig. 4d). Subsequently, SW480 and HCT116 cells were incubated with the cell supernatant of the modified Tregs, and then assessed the cell proliferation, migration and invasion of SW480 and HCT116 cells. As shown in Fig. 5, the cell proliferation, migration and invasion were severely decreased in SW480 and HCT116 cells after treated with cell supernatant of IRF4-overexpressed Tregs (Fig. 5a–c). Thus, these findings showed that IRF4 overexpression promoted the transdifferentiation of Tregs into macrophage-like cells and inhibited cell proliferation, migration and invasion of colon cancer cells.

### IRF4 overexpression promoted the transdifferentiation of tregs into macrophage‐like cells by inhibiting BCL6 expression

**Fig. 6:**
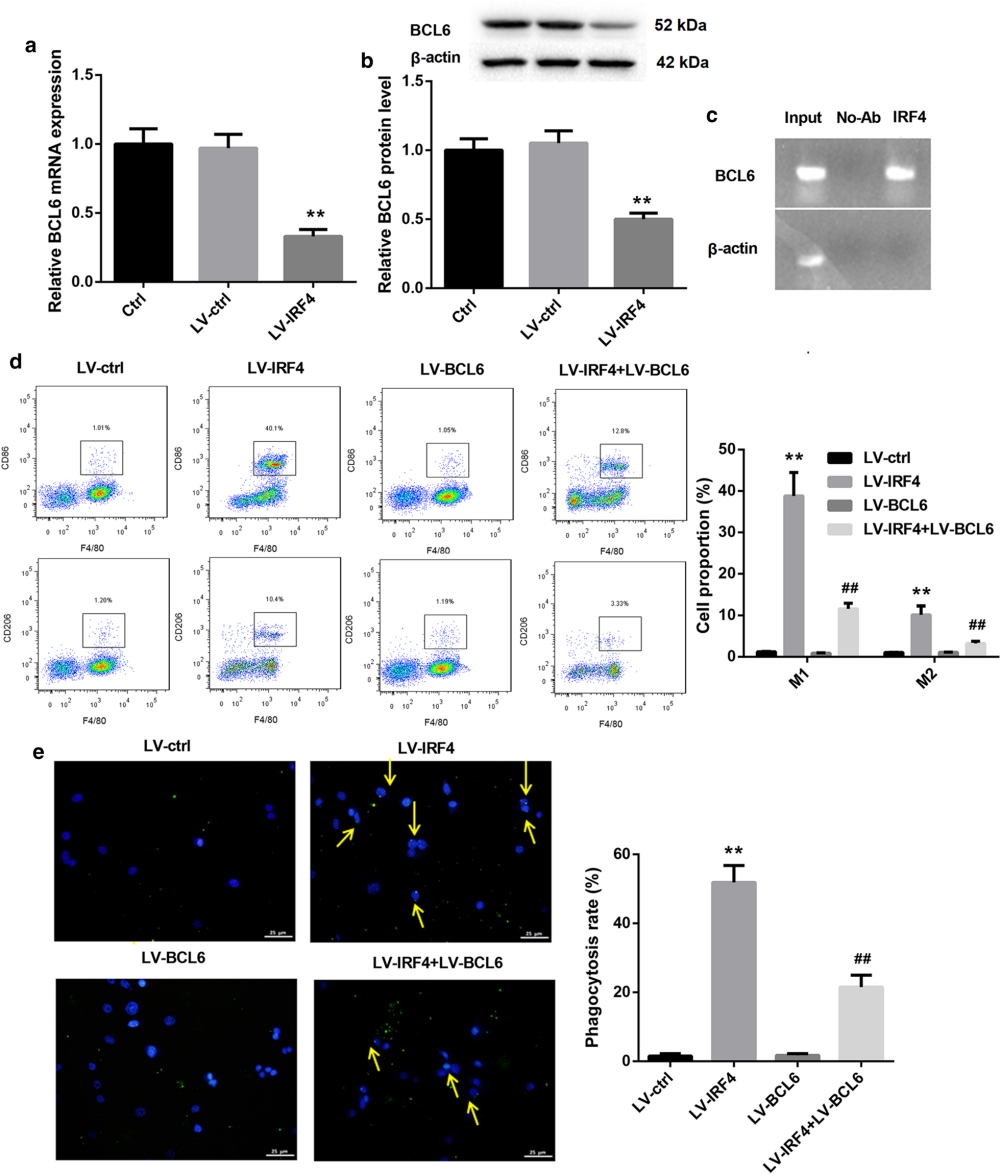
IRF4 overexpression promoted the transdifferentiation of Tregs into macrophage-like cells by inhibiting BCL6 expression. Tregs were transfected with LV-IRF4 or LV-ctrl. Normal Tregs served as control. QRT-PCR (**a**) and WB (**b**) were performed to assess the gene and protein expression of BCL6 in the modified Tregs. **c** The interaction between IRF4 and BCL6 promoter was verified by ChIP. Tregs were transfected with LV-IRF4, LV-BCL6 or LV-ctrl. LV-IRF4 and LV-BCL6 were co-transfected into Tregs. Normal Tregs served as control. **d** The proportions of macrophages in the modified Tregs were detected by flow cytometry. **e** The phagocytosis assay was performed to assess the phagocytosis of the modified Tregs. The phagocytosis positive cells were marked by arrows. (**P < 0.01, versus LV-ctrl; ^##^*P* < 0.01, versus LV-BCL6)

We then determined the molecular mechanisms of IRF4 in regulating the transdifferentiation of Tregs into macrophage-like cells. QRT-PCR and WB data revealed that IRF4 up-regulation led to a down-regulation of BCL6 gene and protein expression in Tregs (Fig. 6a, b). Then, we verified the relationship between IRF4 and BCL6 promoter by ChIP, showing that IRF4 interacted with BCL6 promoter in Tregs (Fig. 6c). Furthermore, Tregs were co-transfected with LV-IRF4 and LV-BCL6, and we evaluated the influence of IRF4 or BCL6 overexpression on the transdifferentiation of Tregs. As shown in Fig. 6d, IRF4 overexpression caused a significant increase in the proportions of M1 and M2 macrophages in Tregs. BCL6 up-regulation had no influence on the polarization of M1 and M2 macrophages in Tregs. However, compared with the Tregs after transfected with LV-BCL6, the proportions of M1 and M2 macrophages were notably enhanced in Tregs in the presence of LV-IRF4 and LV-BCL6 (Fig. 6d). Moreover, IRF4 overexpression significantly promoted the phagocytosis of Tregs, whereas BCL6 up-regulation had no influence on the phagocytosis of Tregs. The phagocytosis was obviously increased in Tregs after co-transfected with LV-IRF4 and LV-BCL6 (Fig. 6e). Thus, these findings indicated that IRF4 overexpression promoted the transdifferentiation of Tregs into macrophage-like cells by inhibiting BCL6 expression.

### Exosomes derived from colon cancer cells down‐regulated IRF4 expression in tregs by transmitting miRNAs

**Fig. 7:**
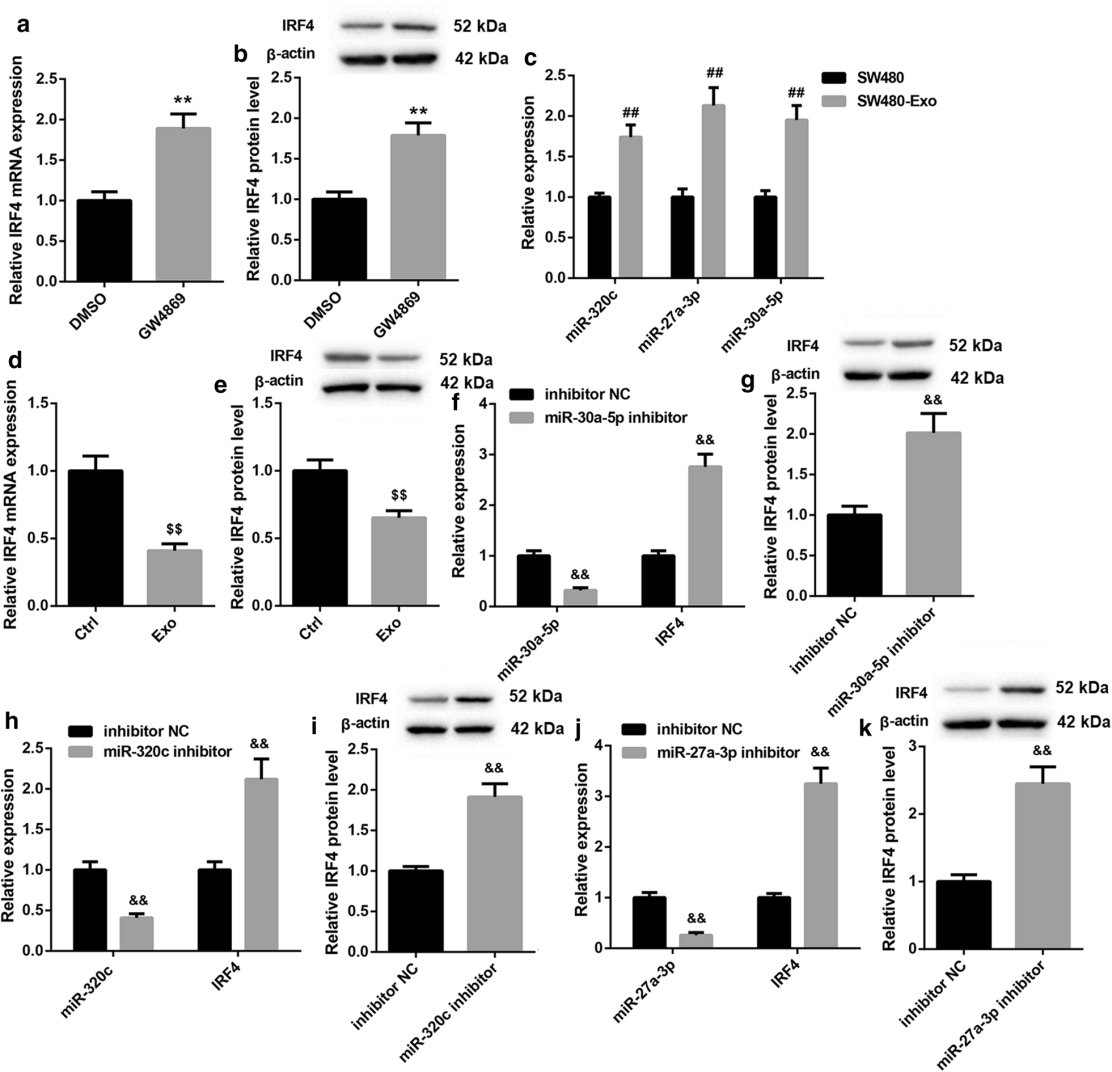
Exosomes derived from SW480 cells down-regulated IRF4 expression in Tregs through miRNAs. SW480 cells and Tregs were co-cultured and incubated with GW4869. QRT-PCR (**a**) and WB (** b**) were performed to assess the gene and protein expression of IRF4 in the Tregs. Exosomes were isolated from SW480 cells. **c** QRT-PCR was performed to detect the expression of miR-320c, miR-27a-3p and miR-30a-5p in the exosomes of SW480 cells. Tregs were incubated with the exosomes of SW480 cells. QRT-PCR (**d**) and WB (**e**) were performed to explore the gene and protein expression of IRF4 in the Tregs. SW480 cells were transfected with miR-30a-5p inhibitor, miR-320c inhibitor, miR-27a-3p inhibitor or the corresponding NC. Then, exosomes were isolated from the modified SW480 cells and incubated with Tregs. **f**,** h** and** j** QRT-PCR was performed to detect the expression of miR-30a-5p, miR-320c, miR-27a-3p and IRF4 in the modified SW480 cells. **g**,** i** and** k** WB was performed to detect the expression of IRF4 in the modified SW480 cells. (**P < 0.01, versus DMSO; ^##^*P* < 0.01, versus SW480; ^$$^*P* < 0.01, versus Ctrl; ^&&^*P* < 0.01, versus inhibitor NC)

We wondered whether colon cancer cells can inhibit the expression of IRF4 in Tregs through secreting exosomes. Co-cultured SW480 cells and Tregs were treated with GW4869 to inhibit exosomes, and GW4869 treatment led to an up-regulation of TRF4 gene and protein expression in Tregs as compared with normal Tregs (Fig. 7a, b). We also found that the expression of miR-320c, miR-27a-3p and miR-30a-5p was significantly up-regulated in the exosomes of SW480 cells with respect to SW480 cells (Fig. 7c). Furthermore, Tregs were incubated with the exosomes of SW480 cells, showing that exosome-treated Tregs exhibited a pronounced down-regulation of of IRF4 (Fig. 7d, e). In addition, SW480 cells were transfected with miR-30a-5p inhibitor, miR-320c inhibitor, miR-27a-3p inhibitor to induce knockdown of miR-30a-5p, miR-320c or miR-27a-3p, and then the exosomes were isolated from the modified SW480 cells to incubate with Tregs. The results obtained from qRT-PCR and WB revealed that miR-30a-5p silencing notably inhibited the expression of miR-30a-5p in Tregs (Fig. 7f). Deficiency of miR-30a-5p enhanced the gene and protein expression of IRF4 in Tregs (Fig. 7f, g). MiR-320c silencing led to a down-regulation of miR-320c, whereas miR-320c deficiency caused an up-regulation of IRF4 in Tregs (Fig. 7h, i). The expression of miR-27a-3p was notably decreased by miR-27a-3p knockdown in Tregs (Fig. 7j). The gene and protein expression of IRF4 was significantly enhanced in Tregs in the presence of miR-27a-3p inhibitor (Fig. 7j, k).

**Fig. 8:**
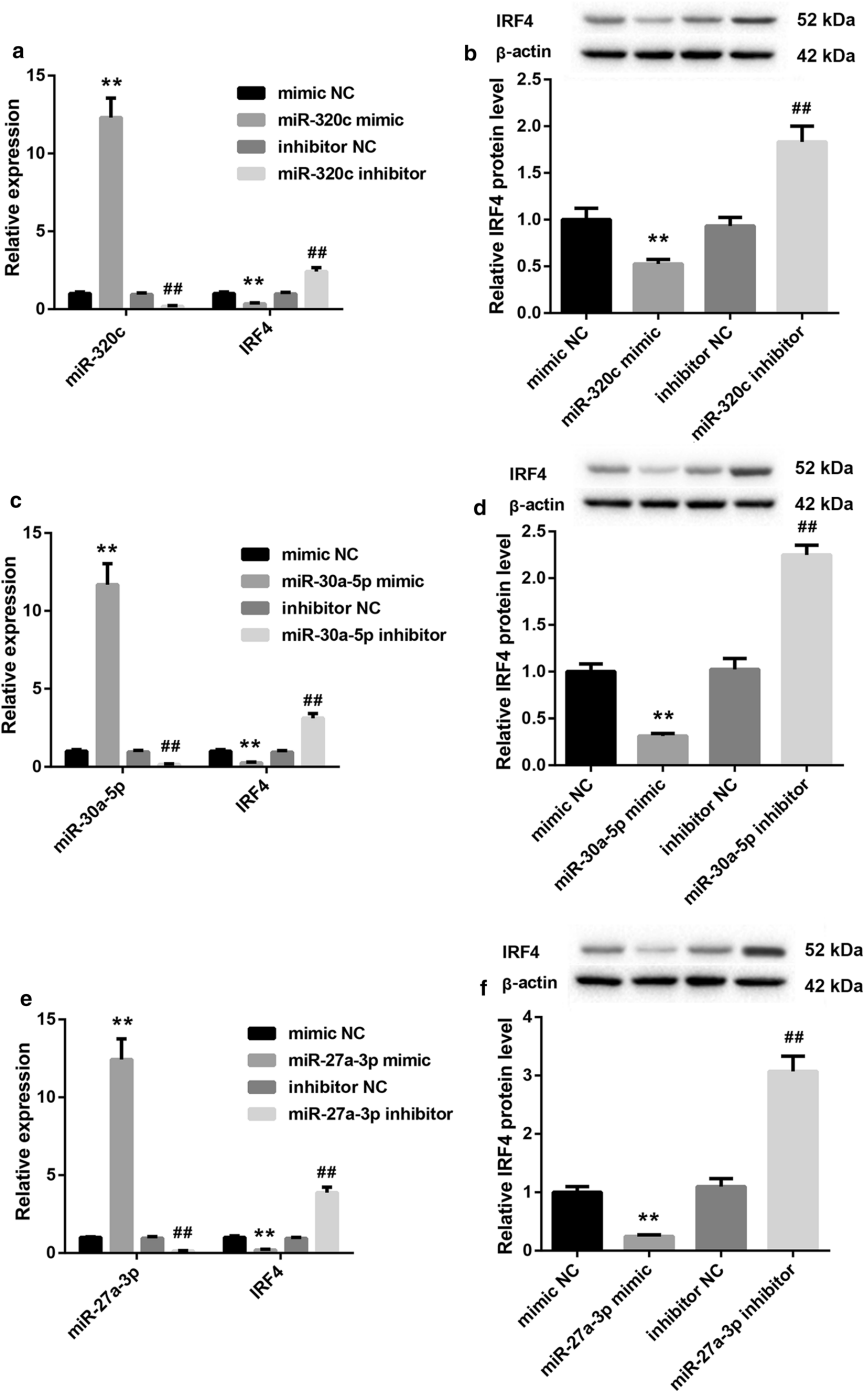
The expression of IRF4 was suppressed by miR-320c, miR-30a-5p and miR-27a-3p. Tregs were transfected with miR-320c mimic, mimic NC, miR-320c inhibitor or inhibitor NC. **a** QRT-PCR was performed to detect the expression of miR-320c and IRF4 in the modified Tregs. **b** WB was performed to explore the expression of IRF4 in the modified Tregs. Tregs were transfected with miR-30a-5p mimic, mimic NC, miR-30a-5p inhibitor or inhibitor NC. **c** QRT-PCR was performed to detect the expression of miR-30a-5p and IRF4 in the modified Tregs. **d** WB was performed to explore the expression of IRF4 in the modified Tregs. Tregs were transfected with miR-27a-3p mimic, mimic NC, miR-27a-3p inhibitor or inhibitor NC. **e** QRT-PCR was performed to detect the expression of miR-27a-3p and IRF4 in the modified Tregs. **f** WB was performed to explore the expression of IRF4 in the modified Tregs. (**P < 0.01, versus mimic NC; ^##^*P* < 0.01, versus inhibitor NC)

**Fig. 9:**
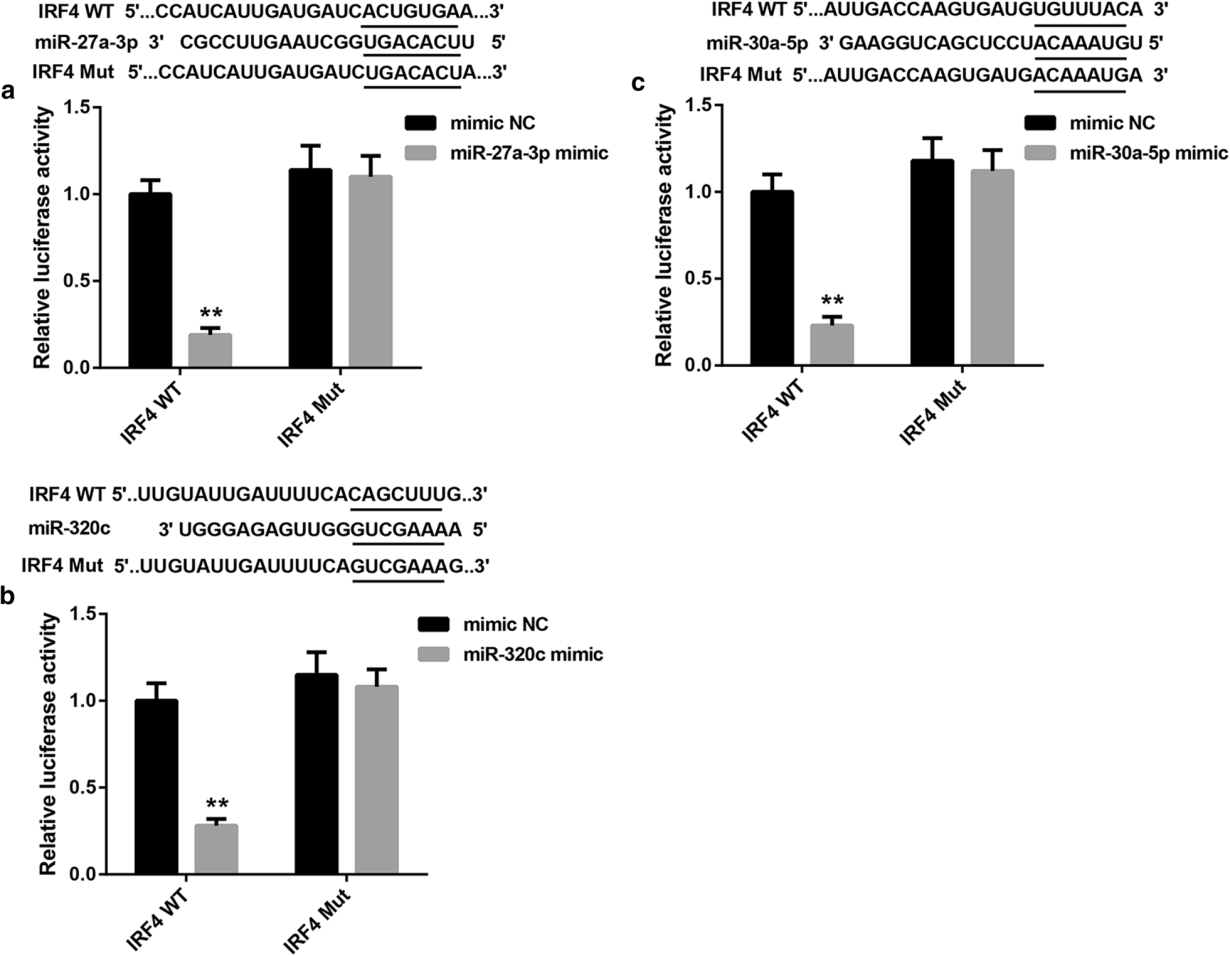
IRF4 was the target of miR-27a-3p, miR-30a-5p and miR-320c, respectively.** a** The Wt (Mut) 3′UTR of IRF4 vector and miR-27a-3p mimic or mimic NC were co-transfected into 293 cells. The interaction between miR-27a-3p and IRF4 was measured by luciferase reporter assay. **b** The Wt (Mut) 3′UTR of IRF4 vector and miR-30a-5p mimic or mimic NC were co-transfected into 293 cells. The interaction between miR-30a-5p and IRF4 was measured by luciferase reporter assay. **c** The Wt (Mut) 3′UTR of IRF4 vector and miR-320c mimic or mimic NC were co-transfected into 293 cells. The interaction between miR-320c and IRF4 was measured by luciferase reporter assay. (**P < 0.01, versus mimic NC)

Next, we up-regulated and down-regulated miR-320c, miR-30a-5p and miR-27a-3p in Tregs respectively, and estimated the effect of these miRNAs on IRF4 expression in Tregs by qRT-PCR and WB. As shown in Fig. 8a, b, miR-320c overexpression promoted miR-320c expression, and the gene and protein expression of IRF4 was repressed by miR-320c overexpression in Tregs. MiR-320c silencing caused a down-regulation of miR-320c in Tregs, and enhanced the gene and protein expression of IRF4 in Tregs (Fig. 8a, b). Moreover, the expression of miR-30a-5p was enhanced by miR-30a-5p up-regulation, whereas miR-30a-5p down-regulation suppressed miR-30a-5p expression in Tregs (Fig. 8c). MiR-30a-5p overexpression significantly inhibited the gene and protein expression of IRF4, whereas the deficiency of miR-30a-5p notably promoted the gene and protein expression of IRF4 in Tregs (Fig. 8c, d). Additionally, miR-27a-3p overexpression enhanced the expression of miR-27a-3p in Tregs. The gene and protein expression of IRF4 was suppressed by miR-27a-3p overexpression in Tregs. The expression of miR-27a-3p was severely inhibited by miR-27a-3p knockdown, whereas the gene and protein expression of IRF4 was highly expressed in Tregs in the presence of miR-27a-3p inhibitor (Fig. 8e, f). In addition, we performed luciferase reporter assay to verify the relationship among miR-320c, miR-30a-5p, miR-27a-3p and IRF4. We found that IRF4 was the target of miR-27a-3p, miR-30a-5p or miR-320c, respectively (Fig. 9a–c).

Taken together, these data suggested that exosomes derived from colon cancer cells down-regulated IRF4 expression in Tregs by transmitting miRNAs.

## Discussion

IRF4 is an essential transcription factor in the immune response of lymphocyte activation and plasma cells to secrete immunoglobulin [24]. IRF4 has been reported to be involved in various cancers. For example, Heimes et al. have found that IRF4 has independent prognostic significance in the node-negative breast cancer, and IRF4 overexpression is associated with the improved outcome of breast cancer [25]. Previous study has confirmed that 8 tag single nucleotide polymorphism in IRF4 are associated with colon cancer [26]. Our data first revealed the biological role of IRF4 in colon cancer. We found that IRF4 was severely down-regulated in the colon cancer tissues. Furthermore, the proportions of Tregs, M1 and M2 macrophages were increased in the colon cancer tissues. Therefore, down-regulation of IRF4 may be associated with the polarization of Tregs in colon cancer.

Our *in vivo* assays further revealed that the inoculation of IRF4-overexpressed SW480 cells effectively attenuated the colon cancer tissue damage, whereas IRF4 knockdown aggravated the colon cancer tissue damage in colon cancer mouse model. Thus, IRF4 overexpression had an inhibiting effect on the progression of colon cancer. Moreover, IRF4 overexpression reduced the proportions of Tregs and M2 macrophages, and enhanced the proportions of M1 macrophages in colon cancer tissues of mice. However, IRF4 silencing caused an increase in the proportions of Tregs and M2 macrophages, and repressed the proportions of M1 macrophages in colon cancer tissues. Tregs play a crucial role in the occurrence of autoimmune diseases and tumors. Previous study has showed that surgical trauma contributes to colon cancer progression by recruiting Tregs to induce an immunosuppressive environment [27]. Tregs frequencies are enhanced in colon cancer patients, and colon cancer may be able to promote recruitment of Tregs as a strategy of immune evasion [28]. Moreover, IRF4 participates in regulating the differentiation and function of Tregs [29, 30]. Thus, we suggested that IRF4 overexpression inhibited colon cancer progression by promoting the transdifferentiation of Tregs into M1 macrophages.

Next, our *in vitro* experiments had confirmed that IRF4 overexpression enhanced the proportions of M1 and M2 macrophages and phagocytosis in Tregs. IRF4 overexpression promoted the transdifferentiation of Tregs into macrophage-like cells. In addition, IRF4 up-regulation significantly suppressed proliferation, migration and invasion of SW480 and HCT116 cells. Taken together, these data demonstrated that IRF4 overexpression inhibited colon cancer by promoting the transdifferentiation of Tregs into macrophage-like cells.

Previous study has reported that BCL6 promotes differentiation of Tregs into Th2 cells by targeting miR-21 [16]. BCL6 regulates the Th2 inflammatory activity of Tregs by suppressing the expression of GATA3 [17]. Thus, BCL6 is closely associated with differentiation of Tregs. In our study, we found that IRF4 repressed BCL6 expression by interacting with BCL6 promoter in Tregs. Furthermore, IRF4 up-regulation enhanced the proportions of M1 and M2 macrophages and phagocytosis in the Tregs. BCL6 up-regulation had no influence on macrophages and phagocytosis of Tregs. The influence conferred by BCL6 up-regulation was partly rescued by IRF4 overexpression. Therefore, these data demonstrated that IRF4 overexpression promoted the transdifferentiation of Tregs into macrophage-like cells by inhibiting BCL6 expression.

It has been reported that exosomes can mediate Tregs immunosuppressive response by transmitting lncRNAs or miRNAs. Recent research has reported that breast cancer-derived exosomes regulate the immunosuppressive functions of CD73^+^γδ1 Treg cells by transmitting lncRNA SNHG16 [31]. MiR-17 in rheumatoid arthritis-derived exosomes may contribute to the pathogenesis of rheumatoid arthritis by repressing Treg differentiation [32]. Our previous study has found that colon cells-secreted exosomal miR-320c, miR-27a-3p and miR-30a-5p interact with IRF4, and inhibit the activity of IRF4 in Tregs. Inactivation of IRF4 abolishes the inhibiting effect of IRF4 on BCL6 expression. Thus, the stable of BCL6 stabilizes the levels of Tregs, and inhibits the anti-tumor immune response. In this work, we found that colon cancer cell-derived exosomes were rich in miR-30a-5p, miR-320c and miR-27a-3p. These exosomal miRNAs severely repressed IRF4 expression in Tregs. Furthermore, futher analysis confirmed that miR-30a-5p, miR-320c or miR-27a-3p repressed IRF4 expression in Tregs by interacting with IRF4. Taken together, our findings showed that exosomes derived from colon cancer cells down-regulated IRF4 expression in Tregs by transmitting miRNAs (Additional file 3: Fig. S3).

## Conclusions

In conclusion, our data demonstrated that IRF4 overexpression promoted the transdifferentiation of Tregs into macrophage-like cells to inhibit the occurrence and development of colon cancer. Thus, our results provide a theoretical basis for an in-depth understanding of the regulatory network in the progression of colon cancer and the development of tumor-targeted drugs.

## Supplementary Information

**Additional file 1: Figure S1.**The efficiency of CD4^+^ T cells selection.CD4+ T cells were isolated from PBMC of peripheral blood of colon cancer patients. Flow cytometry was performed to assess the efficiency of CD4^+^ T cells selection.

**Additional file 2: Figure S2. ** The efficiency of Treg differentiation.CD4^+^ T cells were incubated with TGF-β for Treg differentiation. Flow cytometry was performed to assess the efficiency of Treg differentiation.

**Additional file 3: Figure S3.**The molecular mechanism of IRF4 in colon cancer.

## Data Availability

The datasets used and/or analysed during the current study are available from the corresponding author on reasonable request.

## Abbreviations

ChIP: Chromatin Immunoprecipitation
FBS: Fetal bovine serum
IRF4: Interferon regulatory factor 4
miRNA: microRNA
NC: Negative control
PBMC: Peripheral blood mononuclear cells
qRT-PCR: Quantitative real-time PCR
TAMs: Tumor associated macrophages
Tregs: Regulatory T cells
UTR: Untranslated region
WB: Western blot
Wt: Wild-type
