# Supplementary figures and images for: IRF4 overexpression promotes the transdifferentiation of tregs into macrophage‐like cells to inhibit the development of colon cancer
Source: Cancer Cell Int. 2021 Jan 19;21:58. doi: 10.1186/s12935-021-01766-6 (PMC7816309; doi:10.1186/s12935-021-01766-6)

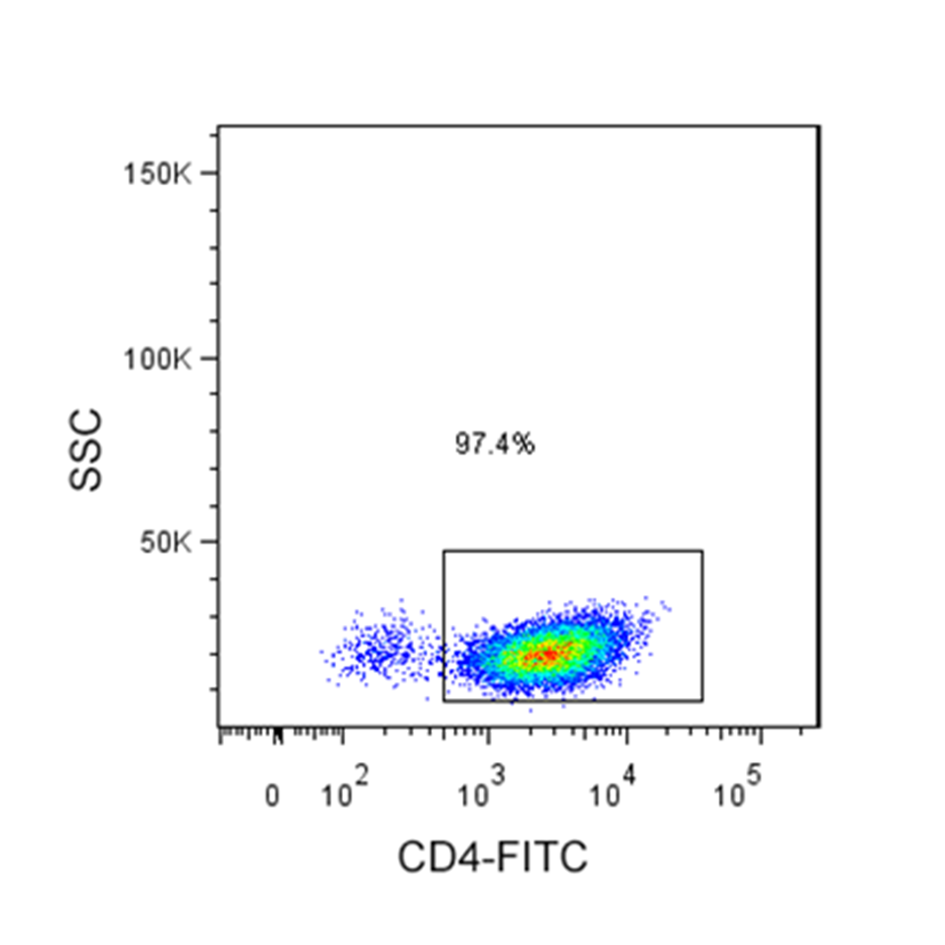

Supplement: Supplementary file 1 — Additional file 1: Figure S1.The efficiency of CD4+ T cells selection.CD4+ T cells were isolated from PBMC of peripheral blood of colon cancer patients. Flow cytometry was performed to assess the efficiency of CD4+ T cells selection. [file 12935_2021_1766_MOESM1_ESM.tif]

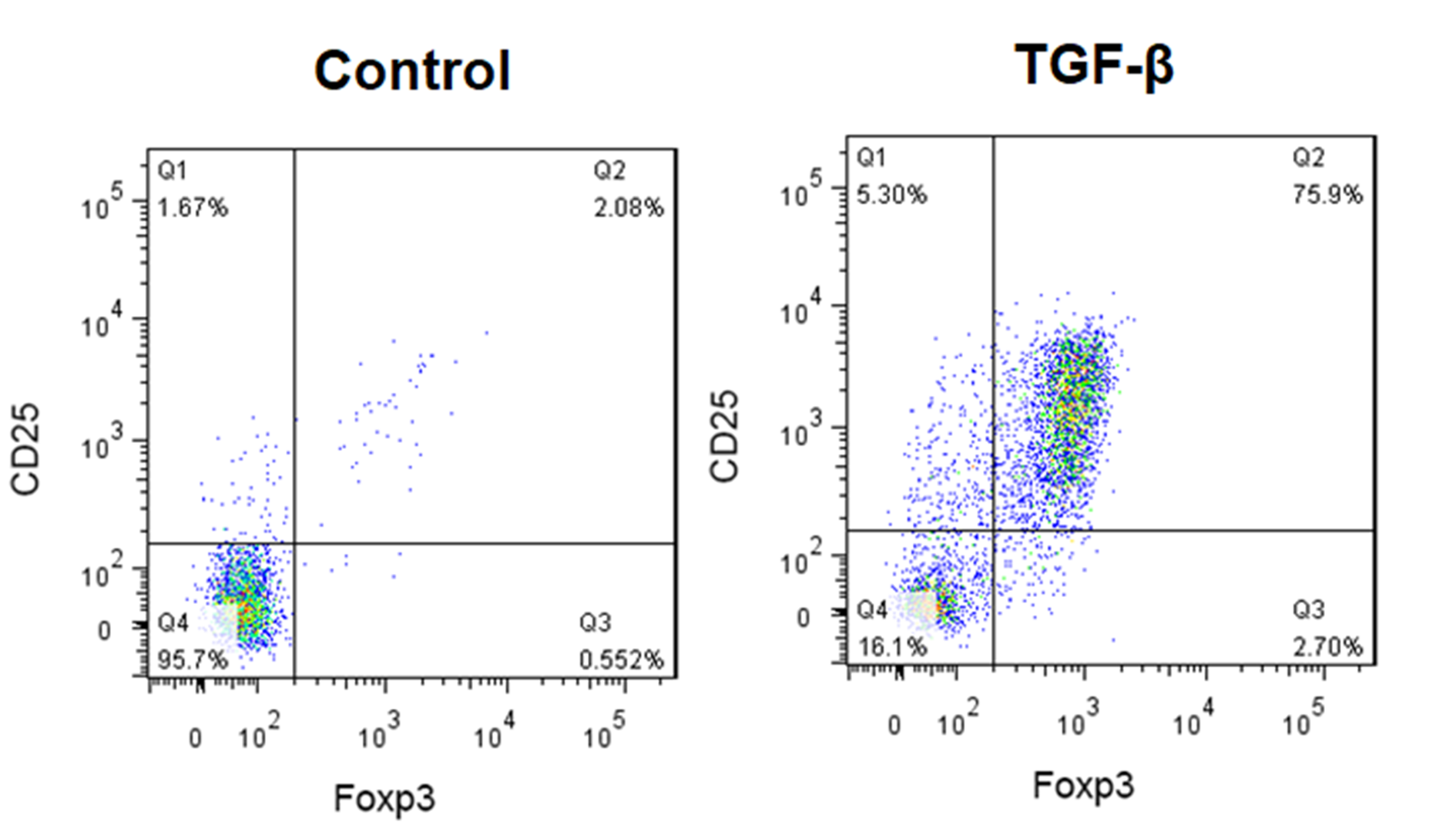

Supplement: Supplementary file 2 — Additional file 2: Figure S2. The efficiency of Treg differentiation.CD4+ T cells were incubated with TGF-β for Treg differentiation. Flow cytometry was performed to assess the efficiency of Treg differentiation. [file 12935_2021_1766_MOESM2_ESM.tif]

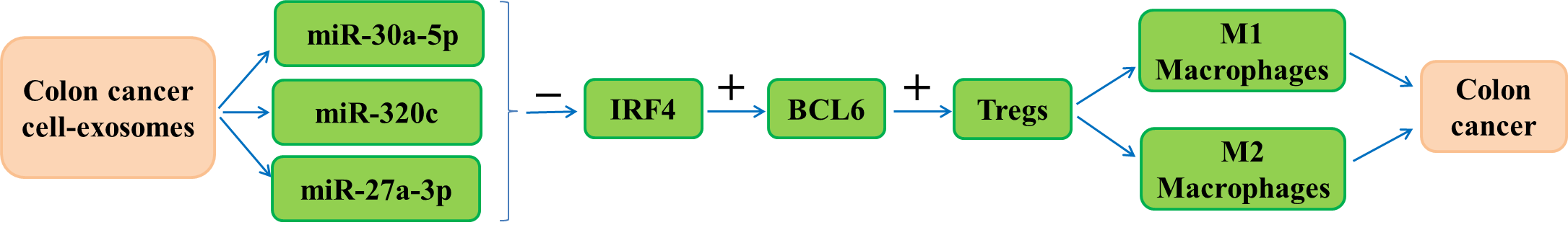

Supplement: Supplementary file 3 — Additional file 3: Figure S3.The molecular mechanism of IRF4 in colon cancer. [file 12935_2021_1766_MOESM3_ESM.tif]
